# Coarse-Grained Molecular Dynamics Modelling of a Magnetic Polymersome

**DOI:** 10.3390/nano8100763

**Published:** 2018-09-27

**Authors:** Aleksandr Ryzhkov, Yuriy Raikher

**Affiliations:** 1 Department of Applied Physics, Perm National Research Polytechnic University, Perm 614990, Russia; 2 Laboratory of Physics and Mechanics of Soft Matter, Institute of Continuous Media Mechanics, Russian Academy of Sciences, Ural Branch, Perm 614013, Russia; raikher@icmm.ru or i.l.raikher@urfu.ru; 3 Department of Theoretical and Mathematical Physics, Institute of Natural Sciences and Mathematics, Ural Federal University, Ekaterinburg 620089, Russia

**Keywords:** magnetic nanoparticles, magnetic polymersomes, micromagnetomechanics

## Abstract

A coarse-grained molecular dynamics framework is proposed to investigate the equilibrium structure and quasi-static deformational response of a magnetic polymersome, a hollow object whose magnetoactive part is its shell (membrane). In the developed scheme, the shell is modelled as a pair of two concentric interfaces, between which a layer of a linearly viscous fluid filled with magnetic nanoparticles is confined; the thickness of this layer slightly exceeds the nanoparticle diameter. The shell boundaries possess weak bending elasticity, very high surface tension and are impermeable for the nanoparticles. The nanoparticles bear permanent magnetic moments and are translationally and rotationally free inside the layer. The factors favoring the particle aggregation are the magneto-dipole coupling and Zeeman interaction with the external field; the impeding factors are thermal motion and steric restrictions imposed by the boundaries. The volume content of magnetic phase in the shell is sufficiently small (below 11 vol.%) to enable one to clearly observe structure patterns occurring in the basic state and under an applied magnetic field. As shown, both the particle concentration and the level of interparticle interaction strongly affect the extent and type of the aggregation that, in turn, causes overall deformation of the polymersome: stretching along the applied field and shrinking in the transverse plane.

## 1. Introduction. Magnetic Polymersomes

The science of magnetic nano-objects in recent decades has been developing at high rate due to their prospects, first of all, in nanomedicine and theranostics. By now, the nomenclature of those entities is really vast including microferrohydrogels, magnetic liposomes, magnetic micelles, magnetic vesicles, and a great many others. In view of that, in order not to confuse the subject of the present work with other similar ones, we begin with definitions. The more so that *magnetic polymersomes* or *magnetopolymersomes* (MPSs) is not yet a customary term in comparison with: (1) *magnetosomes*, and (2) *magnetic endosomes* often encountered in the literature on magnetic soft matter. The objects of type (1) are the iron oxide nanograins (their size in the range of 20–40 nm) synthesised by pelobiont magnetotactic bacteria [1]. Therefore, magnetosomes are completely biogenic entities. They self-organise in chain-like aggregates inside the cytosol, thus making the magnetotactic bacteria sensitive to terrestrial field. Such a “compass” is vital for their normal life. The objects of type (2) are also biogenic: those are small vesicles formed by the cell membrane in the process of endocytosis (uptake) of magnetic nanoparticles from the surrounding medium [2,3]. As the content of endosomes is magnetic, their motion inside the cytosol could be put under external control [4].

Among the artificial magnetically controlled microcontainers, a more subtle but important difference could be established between MPS’s and *magnetic colloidosomes* [5,6] which are microcapsules whose inner content is surrounded by a nanosize magnetically active shell (membrane). In both types of objects the membrane builds up in result of self-assembling of amphiphilic diblock co-polymer molecules and is modified by embedding their magnetic nanoparticles. The difference is that in colloidosomes the nanoparticles are chemically bonded to the membrane, whereas in MPSs, due to appropriate functionalisation of the particles, even though they are confined inside the hydrophobic shell layer, they retain their ability to move inside it [7,8,9,10].

The above-mentioned structure details entail the differences in the object responses to an external field. In colloidosomes (microcapsules) the magnetic nanoparticles are “glued” to their sites within the shell. Because of that, when the interparticle dipole-dipole forces are modulated by an external field and the particles are compelled to regroup, each particle has to entrain in this motion its local polymer environment.

The structure of the MPS membrane could be presented as follows. In an MPS with the overall size (diameter) $D_{out}∼150$ nm) [10], the nanoparticles of diameter a∼10 nm are confined in a closed thin layer made of the hydrophobic tails of the polymer molecules whose polar heads form the inner and outer interfaces of the MPS’s membrane. As the particles are covered with an appropriate surfactant, they are not chemically bonded to the hydrophobic “fluid” and are free to diffuse through it. The only restriction on that motion stems from the fact that the thickness of the intra-membrane layer but slightly exceeds the size of a nanoparticle, and so it should rather resemble not 3D but 2D diffusion.

Had the particles been non-magnetic, the thermofluctuational motion would have resulted just in additional isotropic osmotic pressure acting on the membrane boundaries. For magnetic particles the situation is different. First, the particles interact with each other via dipole-dipole potential and tend to form chain-like aggregates; second, under an applied field $\vec{H}$ the chains unwind and strive to align. Taking $\vec{H}$ as a polar axis, one finds that those chains tend to position themselves in meridional direction, thus stretching the MPS along the field. Assuming that the shell is strong enough as to conserve its area—see justification in Section 2.5 below—one finds that this would mean its simultaneous shrinking in the perpendicular direction. This tendency is opposed, however, by two mechanisms. The first one is the high-elasticity (entropy) resistance of all the polymer component of the membrane. Another mechanism, working to the same effect, is the consequence of anisotropic nature of the magnetostatic interaction and requires that every two magnetised chains set in parallel repel each other in the lateral direction. Therefore, in the confined intra-membrane geometry, the neighbouring meridional nanoparticle chains would strive to move away from one another and, thus, to shrink the MPS in direction transverse to the field. For completeness, we note also an exception from this rule. When the inter-chain distances are very short and the chains are positioned in a “zipper” pattern with respect to one another, their lateral interaction might turn into attraction [11]. However, such a case should really matter only for the systems with high filling factor, and this is not the case for the situations discussed below.

The above-presented considerations, even though providing a general qualitative view, cannot predict the overall deformation of a particular MPS, neither sign nor its magnitude. As follows, those characteristics would depend on the material parameters of a real system: the nanoparticle substance, size and concentration as well as on the polymer shell deformational properties.

As an MPS is a small soft object comprising a countable number of nanoparticles, the coarse-grained molecular dynamics (MD) turns out to be a very appropriate modelling tool to study its basic state and field-induced equilibrium deformation behaviour. Such an approach is successfully used to investigate similar systems, where the magnetic nanoparticles are embedded in the bulk of a loose polymer mesh (ferrogel). The structural elements of the mesh (their scales are different from that of the particles) are described in various ways. For example, in References [12,13,14] each polymer string that connects neighbouring magnetic particles is a sequence of beads (blobs) linked by springs. Another approximation [15] is more close to continual representation and presents the elastic medium as a network (triangular or other), in which the nodes are coupled to each other by entropic springs subjected to some spatial and angular constraints. Here both the nanoparticles and the blobs are the entities of finite size, to which the potentials excluding their overlaps are ascribed. Considering this way as a most prospective, we approximate the magnetic phase of an MPS as an assembly of structureless particles confined in a layer between two concentric borders made of a polymer (in the above-mentioned model sense). Those borders are impermeable for the particles but deform in response to the pressure generated by the latter.

## 2. Problem Statement

Basically, the framework for the problem and algorithms of simulation used in below are taken from the toolbox provided by the ESPReSso package [16] that is specially designed for soft matter problems.

### 2.1. Geometry Construction of the Model

To build the MPS membrane, we adapt the mesh-based model of a 3D closed surface that was proposed in References [17,18] to simulate red blood cells, capsules and alike objects with elastic membranes. In this approach, the surface of a predefined equilibrium shape is covered by a uniform triangular mesh, where each node is a blob in the above-mentioned sense of the coarse-grained MD. The set of inter-blob interactions is defined in such a way that any deviation from the equilibrium geometry induces elastic restoring action of the mesh. The forces are the following: (i) a center-to-center force in each pair of blobs of a triangle that arises as soon as the inter-blob distance deviates from its equilibrium value; (ii) the forces which impede perturbations of the equilibrium angles between the planes of adjacent triangles, they are applied to their vertices. As well, it is possible to introduce (iii) a potential that generates restoring forces which strive to conserve either the local area of every triangle or (iv) the global area of the meshed shape. We remark that the model [17,18] also admits some other variants, for example, (v) the restoring forces could be adjusted to conserve the sample volume.

In our simulations, to make a model MPS softer and more flexible, the bending force and the global area conservation were neglected, whereas to the inter-blob force and the force responsible for the local area conservation relatively small values were ascribed. As well we fully disable the volume conservation mechanism assuming that under equilibrium deformations, the MPS is always in pressure equilibrium with its environment, i.e., the walls are permeable for the low-molecular solvent that occupies both the surrounding and internal spaces of the MPS.

**Figure 1:**
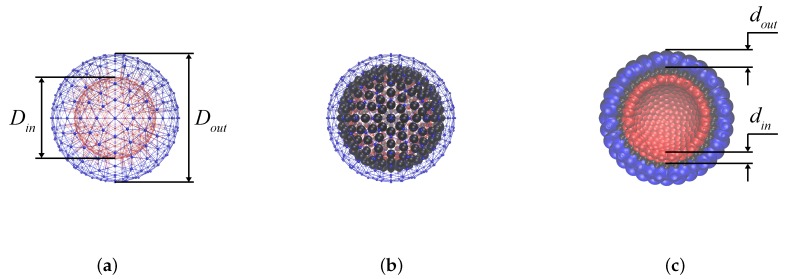
(**a**) outside view of the model MPS “skeleton”: a pair of concentric uniformly meshed spherical shells; (**b**) the same as in (**a**) but the inter-membrane space is filled with solid monodisperse magnetic particles; (**c**) cross-section of the model where the nodes are transformed in finite-size blobs, thus confining the magnetic nanoparticles.

The steps of constructing the model MPS are schematically presented in Figure 1. First, a set of two concentric 3D mesh “skeletons” is built, see Figure 1a with diameters $D_{out}$ and $D_{in}$. The number of nodes in both constructions is equal; and the corresponding nodes are connected by elastic springs in order to exclude too large fluctuations of the inter-surface distance from its equilibrium value *ℓ* in the radial direction. The value of *ℓ* is chosen in such a way that after ascribing finite sizes to the mesh nodes (blobs) viz. $d_{out}$ and $d_{in}$, the remaining space is able to accommodate a monolayer of magnetic nanoparticles of given diameter *a*. Second, several hundred of those nanoparticles are uniformly distributed inside the inter-mesh layer (Figure 1b). In full description, the particles sterically repel one another and each one possesses a built-in magnetic moment $\vec{\mu }$ of a given value μ. However, at this very step of constructing, the interparticle magnetic interaction is not yet turned on.

To avoid penetration of nanoparticles through the shells a soft sphere interaction between dipolar spheres and the nodes of outer and inner boundaries is introduced. Effectively, that means that, with respect to the nanoparticles, the nodes (blobs) appear as the spheres of diameters $d_{out}$ and $d_{in}$, respectively. Due to that, the distances between any adjacent blobs never become sufficient to allow a nanoparticle to squeeze past them. In result, the thickness of the intra-membrane layer accessible for nanoparticles is $\ell =\frac{1}{2}\left(D_{out}-d_{out}-D_{in}-d_{in}\right)$; the cross-section of the model MPS constructed according to the above-presented rules is shown in Figure 1c.

### 2.2. Forces in the Model

On accomplishing the geometry constructing procedure, the interactions which govern the model are introduced. For that, first, all the structure elements of the system, both blobs and nanoparticles, are enumerated sequentially, and all the triangles making the “facets” of the bounding surfaces are also ascribed their individual numbers. A current value of radius-vector ${\vec{r}}_{i}$ of *i*-th particle is obtained from numerical solution of Newton Equation with additional terms corresponding to coupling with Langevin thermostat [19]:

(1) $$m_{i}\frac{d^{2}{\vec{r}}_{i}}{dt^{2}}={\vec{F}}_{i}-\zeta \frac{d{\vec{r}}_{i}}{dt}+{\vec{f}}_{i}\left(t\right),$$

where $m_{i}$ is the particle mass, and the two last terms render, respectively, the dissipative force with the translational friction coefficient that in the Stokes approximation is ζ=3πaη, where η is the viscosity of the fluid. The random force ${\vec{f}}_{i}\left(t\right)$ renders the effect of thermal fluctuations and has the statistics of white noise:

$$\langle {\vec{f}}_{i}\left(t\right)\cdot {\vec{f}}_{j}\left(t^{\prime }\right)\rangle =2\zeta k_{B}T\delta_{ij}\delta \left(t-t^{\prime }\right);$$

here $k_{B}$ is Boltzmann constant, *T* absolute temperature, $\delta_{ij}$ Kronecker delta, and $\delta \left(\cdot \right)$ the Dirac delta function.

Depending on the type of an element of the system, whether it is a blob or a magnetic nanoparticle (MNP), the acting force equals either ${\vec{F}}_{i}={\vec{F}}_{i}^{\mathrm{shell}}$ or ${\vec{F}}_{i}={\vec{F}}_{i}^{\mathrm{MNP}}$, respectively. For a blob, the force ${\vec{F}}_{i}$ is

(2) $${\vec{F}}_{i}^{\mathrm{shell}}=\sum_{j,\;j\neq i}^{Z_{b(i)}}{\vec{F}}_{s,\;ij}+\sum_{k}^{Z_{t(i)}}{\vec{F}}_{\mathrm{al},\;ik}+{\vec{F}}_{\mathrm{Hook},\;i}-\sum_{m,\;m\neq i}^{Z_{\mathrm{MNP}}}\nabla U^{\mathrm{LJ}}\left(r_{im}\right),$$

where $Z_{b}\left(i\right)$ is the set of numbers of blobs connected to *i*-th vertex, $Z_{t}\left(i\right)$ is the set of numbers of the triangles for which *i*-th vertex is a common one, and $Z_{\mathrm{MNP}}$ is the set of MNP numbers.

The center-to-center force acting on *i*-th blob on the part of *j*-th one is described by the neo-Hookean elasticity law:

$${\vec{F}}_{s,\;ij}=k_{s}\frac{{\left(r_{ij}/r_{0,ij}\right)}^{0.5}+{\left(r_{ij}/r_{0,ij}\right)}^{-2.5}}{\left(r_{ij}/r_{0,ij}\right)+{\left(r_{ij}/r_{0,ij}\right)}^{-3}}\frac{r_{ij}-r_{0,ij}}{r_{0,ij}}{\vec{n}}_{ij},$$

where $k_{s}$ is elastic constant, $r_{ij}$ current distance between *i*-th and *j*-th particles, $r_{0,ij}$ equilibrium distance between them, $n_{ij}$ is unit vector connecting the centers of *i*-th and *j*-th particle.

The force which acts on *i*-th blob due to perturbations of the area of *k*-th triangle is:

$${\vec{F}}_{\mathrm{al},\;ik}=-k_{\mathrm{al}}\frac{S_{k}-S_{0,k}}{S_{k}}{\vec{w}}_{ik},$$

where $k_{\mathrm{al}}$ is the area constraint coefficient, $S_{k}$ and $S_{0,k}$ are current and equilibrium areas of *k*-th triangle, respectively; ${\vec{w}}_{ik}$ is unit vector from centroid of *k*-th triangle to *i*-th vertex.

The elastic force acting on *i*-th blob of a given shell on the part of $i^{\prime }$-th blob of another shell (the correspondence between *i* and $i^{\prime }$ is defined at the start of simulation) is taken in a simple harmonic form

$${\vec{F}}_{\mathrm{Hook},\;i}=k_{s}\left(r_{ii^{\prime }}-r_{0,ii^{\prime }}\right){\vec{n}}_{ii^{\prime }},$$

where $k_{h}$ is elastic constant, $r_{ii^{\prime }}$ and $r_{0,ii^{\prime }}$ are current and equilibrium distances between *i*-th and $i^{\prime }$-th particles, respectively; $n_{ii^{\prime }}$ is unit vector directed from the center of *i*-th particle to that of $i^{\prime }$-th one.

The last term in Equation (2) corresponds to the sum of forces emerging from soft repulsion between blobs and MNPs. They are calculated as a gradient of Weeks-Chandler-Andersen (truncated Lennard-Jones) potential [20]:

(3) $$U^{\mathrm{LJ}}\left(r_{im}\right)=\left\{\begin{array}{cc} 4\varepsilon \left[{\left(\frac{\sigma }{r_{im}}\right)}^{12}-{\left(\frac{\sigma }{r_{im}}\right)}^{6}+c_{\mathrm{shift}}\right], & 0<r_{im}<R_{\mathrm{cutoff}},\\ 0, & r_{im}⩾R_{\mathrm{cutoff}}, \end{array}\right.$$

with $r_{im}$ being current distance between the centers of *i*-th and *m*-th particles, ε and σ being energy parameters of the Lennard-Jones potential and $c_{\mathrm{shift}}$ its shift parameter. The cut-off radius $R_{\mathrm{cutoff}}$ equals half the sum of diameters of interacting particles: $\frac{1}{2}\left(d_{i}+d_{j}\right)$ that, in turn, yields $\sigma =1/\sqrt[{6}]{2}R_{\mathrm{cutoff}}$. Therefore, it is potential (3) that defines the sizes of structure elements of the model and ensures their mutual repulsion. As mentioned, the diameters of blobs in both bounding surfaces are chosen in such a way as to confine all the nanoparticles inside the MPS membrane.

The magnetostatic force acting on each magnetic nanoparticle is

(4) $${\vec{F}}_{i}^{\mathrm{MNP}}=-\nabla U_{i}^{\mathrm{MNP}},$$

where

(5) $$U_{i}^{\mathrm{MNP}}=-\mu_{0}\mu {\vec{e}}_{i}\cdot \vec{H}+\mu_{0}\mu^{2}\sum_{j,\;j\neq i}^{Z_{\mathrm{MNP}}}\left[\frac{\left({\vec{e}}_{i}\cdot {\vec{e}}_{j}\right)}{r_{ij}^{3}}-\frac{3\left({\vec{e}}_{i}\cdot {\vec{r}}_{ij}\right)\left({\vec{e}}_{j}\cdot {\vec{r}}_{ij}\right)}{r_{ij}^{5}}\right]+\sum_{k,\;k\neq i}^{Z(N)}U^{\mathrm{LJ}}\left(r_{ik}\right);$$

with $\mu_{0}$ being the permeability of vacuum and Z(N) the set of numbers of all the other elements of the system, both blobs and MNPs. In Equation (5) the first term stands for the Zeeman energy of magnetic moment $\vec{\mu }=\mu \vec{e}$ in the field $\vec{H}$, the second term renders the magneto-dipole interaction with all other dipoles, and the third term describes the total energy of soft sphere repulsion in the system.

Defining of the potentials and their parameters finalises the statement of the problem. A model MPS prepared in such a way, is placed into a single simulation box (no periodic boundary conditions) and is set in contact with the Langevin thermostat. Integration of Equations (1), i.e., thermalisation of the system, begins from the first step, at which the positions and velocities of all the 2×393 blobs are known as well as those of the MNPs. The amount of MNPs is arbitrary but cannot exceed 393, which condition is imposed by the ESPReSso simulation template that we use. (We remark that with this number of elements the problem requires just moderate computer resources, and several its replicas could be calculated without difficulty.) At this stage, the simulation is carried out with allowance for all the interactions except for the magnetic ones: dipole-dipole and Zeeman. As soon as the preliminary thermalisation is finished, the magnetic interactions are turned on, and the simulation is resumed until the system attains its ground state, i.e., the equilibrium at H=0. The magnetised configurations are obtained from it by gradually increasing the strength of the applied magnetic field.

### 2.3. Geometry Parameters

Before proceeding to the simulation results, it is instructive to comment on the reference dimensional values corresponding to the above-given nondimensional ones. In our simulations all the distances are scaled with a certain unit of length, in terms of which the diameter of the magnetic nanoparticle is a=1.2. In those units, the geometry parameters of the MPS and blobs are, respectively,

$$D_{out}=24,\;D_{in}=16,\;d_{out}=3.1,\;d_{in}=2;$$

so that the nondimensional width of the layer confiding the nanoparticles is ℓ=1.45.

Although we are considering rather a generic than a real MPS, we put our estimates to the range that matches the data reported in [7,8,21] and set a≃10 nm. This provides a recalculation rule, which establishes that the adopted unit of length equals 8.3 nm, and, accordingly, the outer diameter of the MPS is $D_{out}≃200$ nm, the distance between the polar interfaces, i.e., the effective thickness of the membrane is d≃33 nm. With allowance for the diameters of the polymer blobs which the outer and inner borders of the membrane are made of, this renders the thickness of the fluid intra-membrane layer as ℓ≃12 nm. This gap is wide enough for longitudinal motion of a single MNP (or a chain of those) but putting two particles across the gap would require a very large energy that makes such a pattern virtually impossible.

The emerging geometrical estimates are close to those of the experimental MPS’s with respect to the overall size and the dimension of the gap layer accessible to the magnetic particles. As to the amount of magnetic nanoparticles in the membrane, for the modelling we take two values of the magnetic phase volume content defined with respect to the volume of the intra-membrane layer: ϕ=5.5 vol.% and ϕ=11 vol.% that reasonably fits the experimentally quantified range for MPS’s of 100–200 nm size, see Reference [21], one of the few where ϕ is reported.

### 2.4. Magnetic Parameters

A typical material of the magnetic particles is maghemite whose magnetisation at room temperature is $M_{s}≃400$ kA/m. Given that, for the magnetic moment of an MNP one gets $\mu ≃2\times 10^{-19}$ SI units. Then the coefficient establishing proportionality between the dimensional field *H* and the nondimensional Langevin argument $\xi =\mu_{0}\mu H/kT$ is about $6\times 10^{-5}$ m/A; in result, the Langevin argument corresponding to reference field H=80 kA/m is ξ≃5.

The parameter of dipole-dipole interparticle interaction is defined as

(6) $$\lambda =\mu_{0}\mu^{2}/\left(a^{3}\right)kT=\mu_{0}{\left(M_{s}\right)}^{2}{\left(\pi /6\right)}^{2}a^{3}/kT,$$

that for the above-adopted material parameters yields λ∼1. Therefore, in the context of the proposed model, the greater values of λ are attainable either with larger MNPs or if the ferromagnetic material has higher magnetisation. Hypothetically, if to replace maghemite by iron ($M_{s}≃2000$ kA/m), the reference value of λ would be about five times greater.

### 2.5. Surface Tension

As mentioned, in our simulations the surface tension of the membrane boundaries confining MNPs is set to be virtually infinite. We base this approximation on the following. The wall thickness of polymersomes ranges 10–30 nm, and the surface tension $\sigma_{s}∼20\;\mathrm{pN}/\mathrm{nm}=2\times 10^{-2}$ N/m [22]. If to compare the energy $\sigma_{s}a^{2}$ required to extend the MPS surface by the area of a particle cross-section (to make the hole for a MNP to run away) with thermal energy of the particle, one gets $\sigma_{s}a^{2}/kT∼10^{3}$ establishing that a non-magnetised particle cannot change the surface area of the MNP.

As to the magnetostatic forces, the energy of a pair of MNPs attracted to one another at the closest distance is about $\mu_{0}\mu^{2}/a^{3}∼\mu_{0}M_{s}^{2}a^{3}$, so that comparison with the energy $\sigma_{s}a^{2}$ required to make a hole in the boundary, yields

$$\sigma_{s}a^{5}/\mu_{0}\mu^{2}∼\sigma_{s}/\left(\mu_{0}M_{s}^{2}a\right)∼10^{2},$$

pointing out that in the magnetic scale of the problem the surface tension of an MPS wall is very high as well. In this connection, we note that the same assumption of conservation of the surface area (high $\sigma_{s}$) was used by authors of Reference [6] in their studies of magnetic colloidosomes.

## 3. Results and Discussion

**Figure 2:**
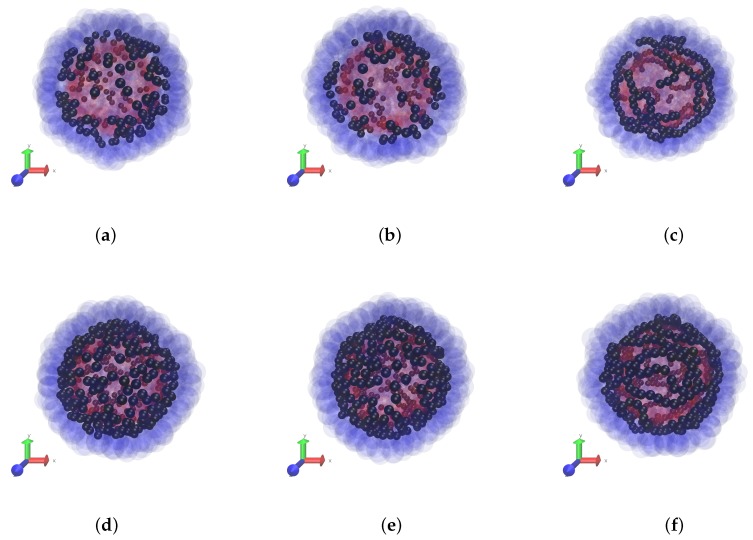
Zero field case. Snapshots of the particle distribution in the MPS membrane at different volume fractions: 5.5 vol.%—panes (**a**–**c**) and 11 vol.%—panes (**d**–**f**); different values of the magneto-dipole interaction parameter: λ=1—panes (**a**,**d**), λ=3—panes (**b**,**e**), λ=5—panes (**c**,**f**).

In this paper we keep the elastic properties of the polar layers (boundaries) of the membrane fixed, and vary the magnetic characteristics of the MPS: the interparticle dipole-dipole interaction and the concentration of the particles. The snapshots of Figure 2 give a clear notion of the initial magnetic structures inherent to non-magnetised MPSs with different intensities of the interparticle interaction λ for two fixed volume concentrations ϕ of the particles, see series of panes (a–c) and (d,e). We remark that clotting of the particles at the periphery of the snapshots is illusional and is due simply to the fact that there the line of view is tangential to the MPS surface.

Strictly speaking, the change of λ corresponds to either variation of temperature or variation of the magnetic substance (different $M_{s}$), which the particles are made of. Although such a test is difficult (or impossible) to realise experimentally, it is instructive from the theoretical viewpoint as it shows that, depending on λ, the ground (zero-field) states of the MPSs are qualitatively different. At a low-to-moderate dipole interaction (up to λ=3), only short particle chains, di- and trimers, could be distinguished at the snapshots. Note that the enhancement of concentration does not seem to substantially affect the aggregate length, it just increases their number. It is at a high level of interaction (λ=5), when non-directed chains whose contour length is of the order of that of the MPS are clearly visible.

**Figure 3:**
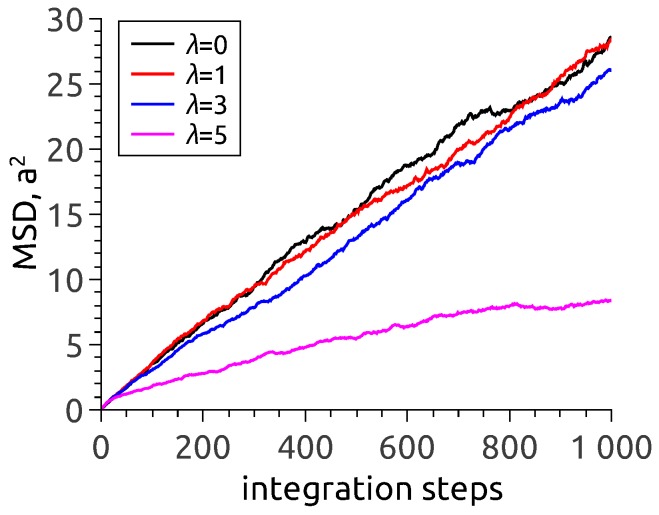
Mean-square displacement of nanoparticles at zero field in the MPS membrane filled to 11 vol.% under variation of the magneto-dipole parameter.

One arrives at the same conclusion when analysing the dependence of mean-square displacement (MSD) of nanoparticles in zero-field situation, see Figure 3. To interpret the data, one has to recall that in an MD simulation with a fixed time step, the number of steps is a direct analog of observation time. As follows from Figure 3, the MSD lines corresponding to λ=0 (no interparticle interaction at all) and λ=1 and 3 virtually coincide and quite well follow the usual free diffusion law $\langle {\left(\Delta \vec{r}\right)}^{2}\rangle \propto t$. It is at λ=5 that the dependence is different. The evident reason is that under those conditions, the particles are by no means isolated: in vast majority they are incorporated in chain aggregates where their motion is to a high extent hindered.

**Figure 4:**
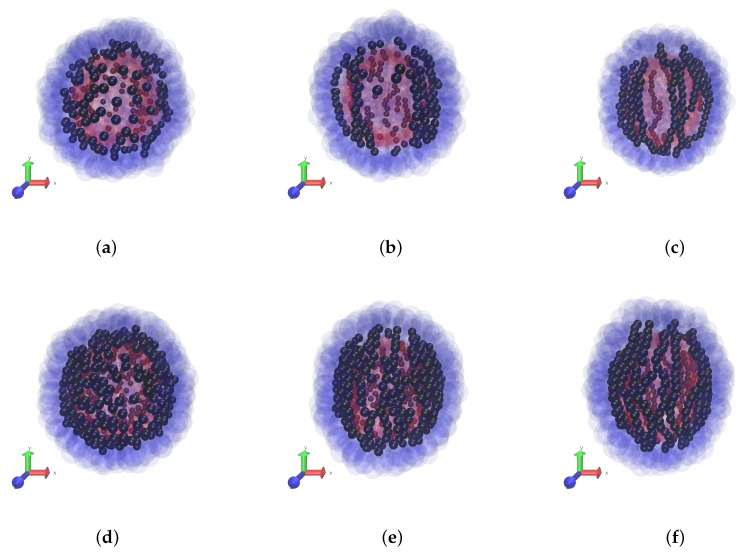
Under field ξ=10. Snapshots of the particle distribution in the MPS membrane at different volume fractions: 5.5 vol.%—panes (**a**–**c**) and 11 vol.%—panes (**d**–**f**); different values of the magneto-dipole interaction parameter: λ=1—panes (**a**,**d**), λ=3—panes (**b**,**e**), λ=5—panes (**c**,**f**).

The effect of interparticle interaction on the magnetodeformational response of the MPS is illustrated by the snapshots of Figure 4, which correspond to the situation under a strong field: ξ=10. We remind that parameter ξ measures the ratio of Zeeman to thermal energy for an MNP. Thus, for ξ≫1, fluctuations of the magnetic moments are relatively weak, and vectors $\vec{\mu }$ of the particles readily align with the field. This strongly enhances their magnetic attraction in the “head-to-tail” direction, i.e., the direction of $\vec{H}$, and this favors appearance of long erect chains. In their striving to full straightening, the chains work against the confinement imposed by the shell and this results in the MPS elongation. This is visible in Figure 4, and, as seen, the elongation increases with the enhancement of the particle concentration and the more so with the magneto-dipole interaction parameter.

As the snapshots show, the emerging chain structure is not perfect, and this could be attributed to several reasons. First, those are kinetic restrictions: too much time is necessary for the already formed long chains to change their conformations. Second, as mentioned, the concept of laterally repulsive particle strings is entirely valid only for the system with very low concentration of the particles. Otherwise, the “zipper”-like attachment of neighbouring chains becomes energetically favorable. The spots where such patterns are present one could easily distinguish in Figure 4, especially in panes (c,f).

**Figure 5:**
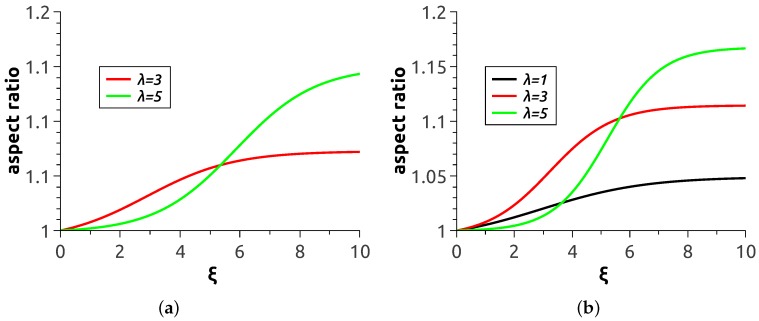
Field strength dependence of the aspect ratio of an MPS under variation of the external field, (**a**) magnetic particle volume fraction ϕ=5.5 vol.%; (**b**) magnetic particle volume fraction ϕ=11 vol.%.

Quantitative characterisation of the magnetodeformational behaviour of MPS’s is presented in Figure 5, where the aspect ratio of the polymersome cross-section by the plane parallel to the direction of $\vec{H}$ is plotted as a function of applied field strength. Each curve there was obtained by averaging over ten realisations of the magnetisation process with the same material parameters and same initial conditions. Expectedly, the greater the magnetic content of the shell and the level of magnetic interaction of the particles the more pronounced is the elongation effect of the field. It should be noted that the presented smooth curves were carried out with the aid of sigmoid fitting of calculation data points. Because of that, the curve corresponding to λ=1 at ϕ=5.5 wt.% is not presented: for it, the set of ten realisations does not suffice to reduce the uncertainty to a reasonable level.

All the presented curves display the tendency to saturation. The cause of that is clear: as soon as all magnetic moments in a chain are aligned with the field (at ξ≫1), the magnetostatic force that the chain generates ceases to grow. However, at intermediate stages of magnetisation, the system undergoes complex structure evolution. For example, the non-directed chains in Figure 2e are apparently shorter than those in Figure 4e, whereas comparison of respective panes (f) of the same Figures reveals the opposite tendency. In our view, the rules governing such rearrangements could hardly be derived analytically. Indeed, to evaluate the number of the emerging chains as well as their length and curvature one has to carry out minimisation of the total (magnetic + elastic) energy of a multi-particle assembly subjected to such a complicated restriction as closeness of the layer and conservation of the areas of its surfaces. Therefore, numerical experiment seems the only adequate way to investigate MPS’s.

The main issue of interest is the overall deformation of an MPS as it determines its ability to work in numerous previewed applications. As Figure 5 shows, the aspect ratios attainable with the model are about 1.17 maximum. This is much lower than those (about 2) for experimentally prepared large-size (about 500 μm) magnetic colloidosomes reported in [6]. To understand the difference, we recall the results of References [23,24] where a continuum model of an MPS has been studied. It has been shown that the aspect ratio of a ferrovesicle in a given field grows substantially as the ratio of the shell thickness to the overall size of the vesicle goes down. For illustration, we remark that In Reference [6] this ratio was about 1/50 whereas for the present model, see Equation (6) it is $\left(D_{out}\right)-D_{in}/2D_{out}$ = 1/6, i.e., in our case is about ten times greater. Given that, the difference in the attained aspect ratios is quite understandable.

We remark, however, that comparison of the continuum model to the present one could not be other than qualitative. From structural viewpoint, the essence of the continuum approach is that local magnetic properties of any part of the membrane do not depend on the applied field. On the contrary, in the MD model of MPS, application of the field strongly affects the spatial distribution of the magnetic particles.

Finally we remark that the family of magnetic polymersomes is growing, and these objects are getting specialised for particular purposes. In this connection, we point out the idea to modulate permeability of the shell for the inner content of the MPS (e.g., a drug) with the aid of an applied field either mechanically [6] or via inductive heating [10]. A possible way to that goal, is the effect of field-driven spatial redistribution of nanoparticles in the MPS shell that we demonstrate here.

## 4. Conclusions

Magnetic polymersomes (MPS’s) are micro-objects with high potential impact in applications, the essence of which is the field control over their structure and deformational behaviour. Physically, the MPS’s are complex systems with a hierarchy of their intra- and inter-component interactions. Due to the presence of that many degrees of freedom, analytical description of MPS’s basic state and field-dependent properties cannot go very far. On the other hand, with allowance for the reference magnitudes of their inner and outer scales, MPS’s are very well fit for molecular dynamics modelling.

In this paper we apply one of the schemes of coarse-grained molecular dynamics for a detailed analysis of an isolated MPS. As far as we know, this is the first attempt of such a kind. The object considered here is a model one and as such cannot be directly associated with any real MPS known insofar. However, that is not the purpose of the work. Our goal is to formulate a consistent generic model for the problem in such a way that the model would be well prone for extending and detailing. Here the developed framework is applied to a quite particular situation, where just some magnetic parameters of an MPS with a given size, elasticity, etc. are varied. However, in our view, even this limited example suffices to demonstrate good workability of the molecular dynamics approach and proves its high potential for further studies. There are a number of interesting tasks to be accomplished in order to facilitate the use of the model and to bring it closer to real systems. Firstly, the description of equilibrium properties should be advanced: one needs to have a clear correlation between the potentials coupling the non-magnetic elements and the customary elastic characteristics of the membranes. In particular, the occurred rather low deformability of the studied MPS might be due just to a too high rigidity of the model membrane. As well the pre-simulation scheme should be made more flexible and comprehensible in choosing all the geometry parameters of the MPS’s.

Yet untouched, although quite attainable, is the whole scope of dynamic (kinetic) problems concerning time-dependent response of the MPS’s; there the interplay of viscosity effects would be of vital importance. For example, the assumption that the fluid that fills the membrane and in which the magnetic nanoparticles move through, is a Newtonian one, is evidently very naive. Indeed, the viscoelastic nature of the solution of hydrophobic polymer blocks separating the polar interfaces of the membrane should manifest itself in full when the behaviour of an MPS under a time-varying field comes in question.
